# Impact of hepatic steatosis on liver stiffness measurement by vibration-controlled transient elastography and its diagnostic performance for identifying liver fibrosis in patients with chronic hepatitis B

**DOI:** 10.1186/s13244-024-01857-8

**Published:** 2024-11-22

**Authors:** Zhiyuan Chen, Ye Huang, Yan Zhang, Dongjing Zhou, Yu Yang, Shuping Zhang, Huanming Xiao, HaiXia Li, Yupin Liu

**Affiliations:** 1 Department of Radiology, The Second Affiliated Hospital of Guangzhou University of Chinese Medicine, Guangzhou, China; 2 Second Clinical Medical College of Guangzhou University of Traditional Chinese Medicine, Guangzhou, China; 3 Integrated Department, The Second Affiliated Hospital of Guangzhou University of Chinese Medicine, Guangzhou, China; 4 Department of Pathology, The Second Affiliated Hospital of Guangzhou University of Chinese Medicine, Guangzhou, China; 5 Department of Hepatology, The Second Affiliated Hospital of Guangzhou University of Chinese Medicine, Guangzhou, China; 6 Department of Radiology, Bayer Healthcare Limited Company, Guangzhou, China

**Keywords:** Chronic hepatitis B, Metabolic dysfunction-associated steatotic liver disease, Liver fibrosis, Liver stiffness measurement, Proton density fat fraction

## Abstract

**Objectives:**

To explore the impact of hepatic steatosis measured by MRI-proton density fat fraction (MRI-PDFF) on liver stiffness measurement (LSM) value and its diagnostic performance for staging liver fibrosis in patients with chronic hepatitis B (CHB).

**Methods:**

A total of 914 patients with CHB who underwent liver biopsy and MRI-PDFF were retrospectively reviewed. The influence of MRI-PDFF on LSM value was assessed using univariate and multivariate linear analyses. To assess the influence of liver steatosis on the diagnostic performance of LSM, a series of ROC analyses were performed and compared by stratifying patients into non-steatosis (PDFF < 5%) and steatosis (PDFF ≥ 5%) groups according to MRI-PDFF values. The effects of different LSM cut-off values on the false-positive rate in the steatosis cohort were compared using McNemar’s test.

**Results:**

LSM values were significantly affected by MRI-PDFF in the entire cohort (*B*-coefficient: 0.003, *p* < 0.001), F1 cohort (*B*-coefficient: 0.005, *p* < 0.001), and F2 cohort (*B*-coefficient: 0.003, *p* = 0.002). Hepatic steatosis was not observed to have a significant influence on the ROC curve of LSM for staging liver fibrosis. Compared with using the cut-off values for the CHB cohort, using relatively higher cut-off values for hepatic steatosis significantly improved the false-positive rate of LSM in the steatosis cohort.

**Conclusion:**

Steatosis significantly influenced LSM, with a higher value in the early stage of liver fibrosis but did not affect the diagnostic efficiency of LSM for staging liver fibrosis. Moreover, using relatively high cut-off values significantly improved the false-positive rate of LSM in CHB patients with steatosis.

**Clinical relevance statement:**

The identified correlation between MRI-PDFF and VCTE-measured LSM is not clinically relevant since the diagnostic performance of LSM in staging liver fibrosis is not affected by steatosis. A higher cut-off should be applied in CHB patients with steatosis to improve the false-positive rate.

**Key Points:**

- Steatosis can affect liver stiff measurement (LSM) values in the early stage of liver fibrosis.
- The diagnostic performance of LSM in staging liver fibrosis is not affected by steatosis.
- LSM’s cutoffs should be increased in patients with steatosis to improve the false-positive rate.

**Graphical Abstract:**

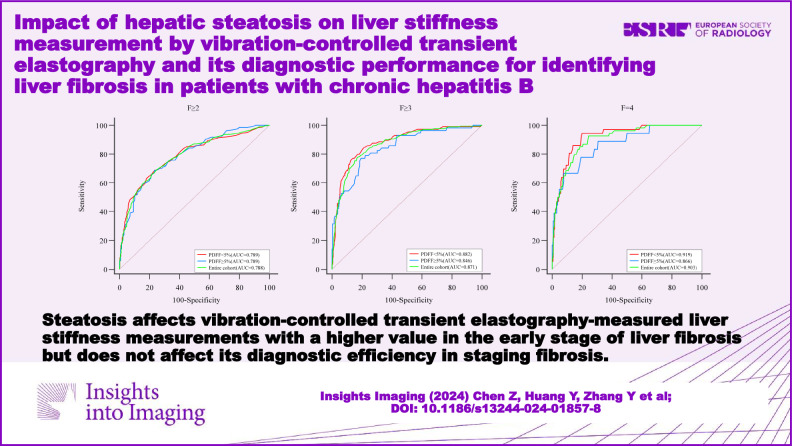

## Introduction

Hepatitis B virus (HBV) infection causes significant morbidity and mortality worldwide [1], and more than one-third of HBV infections and deaths occur in China [2]. HBV infection can lead to chronic hepatitis B (CHB), liver fibrosis, cirrhosis, hepatocellular carcinoma, and death. Staging of liver fibrosis is essential for risk stratification, treatment, and management of patients with CHB [3–5]. Although liver biopsy is considered the gold standard approach for liver fibrosis assessment, it is invasive and associated with pain, high cost, sampling error, and interobserver or intraobserver variability [6]. Therefore, noninvasive diagnostic methods for liver fibrosis staging are more commonly used in clinical practice.

Vibration-controlled transient elastography (VCTE) is the most widely used noninvasive fibrosis assessment method and has been validated in large cohorts [7, 8]. However, the liver stiffness measurement (LSM) value obtained by VCTE is likely to be affected by other conditions, such as acute hepatitis, alcohol abuse, food intake within 2–3 h, congestive heart failure, and extrahepatic cholestasis [9]. Because of the increasing incidence of obesity and metabolic syndrome, concomitant metabolic dysfunction-associated steatotic liver disease (MASLD) has become increasingly common in patients with CHB. Therefore, the impact of hepatic steatosis on the VCTE-measured LSM value and its diagnostic performance in these patients should be identified.

Whether steatosis affects the accuracy of the VCTE-measured LSM in staging liver fibrosis remains unclear, with studies having shown conflicting results [10–15]. Most cohorts evaluated in past studies comprised patients with MASLD, and the conclusions of these studies might not apply to patients with CHB because of the different liver fibrosis scoring systems used in the two diseases. Furthermore, the steatosis assessment tool used in these earlier studies was the controlled attenuation parameter, which is inferior to the MRI-based proton density fat fraction (MRI-PDFF) across the full spectrum of liver fat [16, 17]. To our knowledge, there is no data exploring the impact of hepatic steatosis on the VCTE-measured LSM value and its diagnostic efficiency using the most accurate noninvasive biomarkers of liver fat.

Therefore, this study aims to assess the impact of hepatic steatosis measured by MRI-PDFF on the LSM value, and its diagnostic performance for staging liver fibrosis in patients with CHB.

## Materials and methods

### Patients

The ethics committee of our institution approved this retrospective study, and the requirement for written informed consent was waived. The data of patients who underwent chemical shift-encoded (CSE) MRI and liver biopsy at our institute between January 2017 and November 2023 were retrospectively reviewed. A portion of the subjects (26.26%) participated in the Thirteenth Five-Year Plan for Major and Special Programs of the National Science and Technology of China (data on MRI-PDFF have not been published). All VCTE, MRI examinations, and liver biopsies (including techniques and histology performed for fibrosis, necroinflammation, and steatosis evaluation) were performed for clinical purposes unrelated to this investigation. The inclusion criteria were detectable hepatitis B surface antigen (HBsAg) for ≥ 6 months, age of > 18 years, no history of hepatectomy, and laboratory tests performed within 1 week and VCTE and MRI within 1 month before liver biopsy. The exclusion criteria were coinfection with other hepatitis viruses or human immunodeficiency virus, treatment with hepatotoxic or steatogenic drugs within 1 month before biopsy, alcohol consumption of > 20 g/day for men or > 10 g/day for women within the previous 2 years, incomplete VCTE or laboratory data, liver biopsy specimens of < 15 mm in length, CSE-MRI artifacts that may affect the measurement result, and iron overload in view of remarkably decreased signals on T2-weighted images for two-point Dixon MRI or an R2* value of > 449 s^−1^ in six-point Dixon MRI, which may affect liver fat quantification [18].

### CSE-MRI protocol and measurement of hepatic fat fraction

Patients underwent a multi-echo three-dimensional spoiled gradient echo sequence using two 3.0-T MRI vectors with an 8-channel Atlas SPEEDER phased-array coil (Titan; Canon Medical Systems, Otawara, Japan) or an 18-channel phased-array coil (Prisma; Siemens Healthineers, Erlangen, Germany). All patients fasted for ≥ 8 h before the MRI. The acquisition parameters are shown in Supplementary Methods S1.

After imaging acquisition, in-phase, out-of-phase, fat-only, and water-only images were consequentially reconstructed, and axial fat fraction maps were automatically generated. A senior radiologist who was blinded to the clinical and histological data then drew the region of interest (ROI) on the fat fraction maps to measure the hepatic fat fraction. Six 15-mm-diameter circular ROIs were placed in the right lobe on three central continuous sections (two ROIs per section). To match the liver biopsy location, the ROIs were placed on the liver parenchyma approximately 2 cm from the liver edge, avoiding artifacts, bile ducts, focal lesions, and large vessels (Supplementary Fig. S1). The mean of the six ROI measurements was taken as the patient’s hepatic fat fraction value. All patients were categorized as non-steatosis (PDFF < 5%) or steatosis (PDFF ≥ 5%) based on the MRI-PDFF results [19].

### Clinical and laboratory data

Demographic, anthropometric and clinical data (age, sex, body mass index [BMI], antiviral therapy, hypertension, diabetes mellitus [DM], and impaired glucose tolerance [IGT]) and fasting laboratory indices (platelets, alanine aminotransferase [ALT], aspartate aminotransferase [AST], albumin, alkaline phosphatase, gamma-glutamyl transferase, total bilirubin [TBIL], HBsAg, hepatitis B e antigen [HBeAg], hepatitis B virus DNA (HBV DNA), total cholesterol, total triglyceride, high-density lipoprotein cholesterol, and low-density lipoprotein cholesterol) were collected within 1 week before liver biopsy. The AST-to-platelet ratio index (APRI) and fibrosis-4 index (FIB-4) [20] were calculated for each patient using the following formulas:

$${{{\rm{APRI}}}}={{{\rm{AST}}}}[/{{{\rm{upper}}}}\; {{{\rm{limit}}}}\; {{{\rm{of}}}}\; {{{\rm{normal}}}}]/{{{\rm{platelets}}}}\,[{10}^{9}/{{{\rm{L}}}}]\times 100$$

$${{{\rm{FIB}}}}{\mbox{-}}4={{{\rm{age}}}}[{{{\rm{years}}}}]\times {{{\rm{AST}}}}[{{{\rm{U}}}}/{{{\rm{L}}}}]/({{{\rm{platelets}}}}[{10}^{9}/{{{\rm{L}}}}]\times \sqrt{{{{\rm{ALT}}}}[{{{\rm{U}}}}/{{{\rm{L}}}}]})$$

### LSM

VCTE was performed by a trained hepatologist blinded to the clinical and imaging data using a FibroScan® 502 Touch model (M Probe; Echosens, Paris, France). Patients were asked to fast for ≥ 3 h prior to the examination. VCTE measurements were obtained in the supine position with the right arm fully adducted, by scanning the abdomen at the location of the right liver lobe during a 10-s breath hold. LSM results were considered unreliable if there were < 10 successful measurements, there was a success rate of < 60%, or the interquartile range [IQR]/median was ≥ 30%. After ten consecutive successful measurements, the median valid value was taken as the liver LSM value.

### Histological evaluation

The indications for liver biopsy in patients with CHB were consistent with the American Association for the Study of Liver Diseases guideline [4]. Before providing written informed consent, the patients received full information regarding the indications, risks, and alternatives to liver biopsy. A liver biopsy was performed by a senior hepatologist under ultrasound guidance, and specimens of the right lobe where the right branch of the portal vein enters the liver were obtained using a 16-gauge needle. All specimens were ≥ 15 mm in length and/or contained > 10 portal tracts. The formalin-fixed paraffin-embedded specimens were stained with hematoxylin–eosin and Masson’s trichrome. Light microscope examination was performed by a hepatopathologist with > 10 years of experience who was blinded to the clinical and imaging data. According to the Batts-Ludwig scoring system [21], liver fibrosis (F) was semi-quantitatively evaluated and staged as follows: F0, no fibrosis; F1, mild fibrosis; F2, significant fibrosis; F3, advanced fibrosis; F4, and cirrhosis. Likewise, liver necroinflammatory activity (G) was graded G0 to G4, representing no, minimal, mild, moderate, and severe activity, respectively. The degree of steatosis (S) was categorized on a four-point scale as: S0 (< 5%, none), S1 (5–33%, mild), S2 (33–66%, moderate), and S3 (> 66%, severe).

### Statistical analysis

Data are reported as frequency (percentage) for categorical variables and as median (IQR) for continuous variables. The Mann–Whitney *U*-test or Kruskal–Wallis *H* test with Bonferroni post-test was used, as appropriate, to evaluate between-group differences in continuous variables. The chi-square or Fisher’s exact test was used, as appropriate, to evaluate between-group differences in categorical variables.

The impact of MRI-PDFF on LSM values was evaluated using univariate linear regression analysis in the entire cohort and for each fibrosis stage. A multivariate linear regression model with stepwise forward selection was then constructed with MRI-PDFF, fibrosis stage, necroinflammatory activity grade, sex, age, antiviral therapy, HBeAg status, ALT, TBIL, and BMI as candidate covariates, and LSM as the outcome variable. LSM was Box-Cox transformed to approximate a normal distribution before univariate and multivariate linear regression analyses.

LSM performance was assessed by measuring the area under the receiver operating characteristics (ROC) curve (AUC) for discriminating different stages of fibrosis: significant fibrosis or higher (*F* ≥ 2), advanced fibrosis or higher (*F* ≥ 3), and cirrhosis (*F* = 4). For comparison purposes, ROC curves of APRI, FIB-4, and LSM for discriminating different stages of fibrosis were plotted, and the corresponding AUC values were compared using DeLong’s test. To assess the influence of liver steatosis on the diagnostic performance of LSM, ROC analyses were performed and compared by stratifying patients according to their MRI-PDFF values.

The sensitivity, specificity, false-positive rate (FPR), positive predictive value (PPV), negative predictive value (NPV), and accuracy of LSM were determined using cut-off values according to the systematic review by Duarte-Rojo et al (for the CHB cohort: 7 kPa for *F* ≥ 2, 8 kPa for *F* ≥ 3, and 11 kPa for *F* = 4; for the hepatic steatosis cohort: 7 kPa for *F* ≥ 2, 10 kPa for *F* ≥ 3, and 13 kPa for *F* = 4) [22]. The sensitivity, specificity, FPR, PPV, NPV, and accuracy of LSM for discriminating different stages of fibrosis in the non-steatosis and steatosis cohorts were compared using the McNemar’s test, the chi-square test, or Fisher’s exact probability test as appropriate.

Statistical analysis was performed using SPSS version 26.0 (IBM Corp., Armonk, NY, USA) and MedCalc version 20.0 (MedCalc, Ostend, Belgium). A two-sided *p* value of < 0.05 was considered statistically significant.

## Results

### Patients

**Fig. 1:**
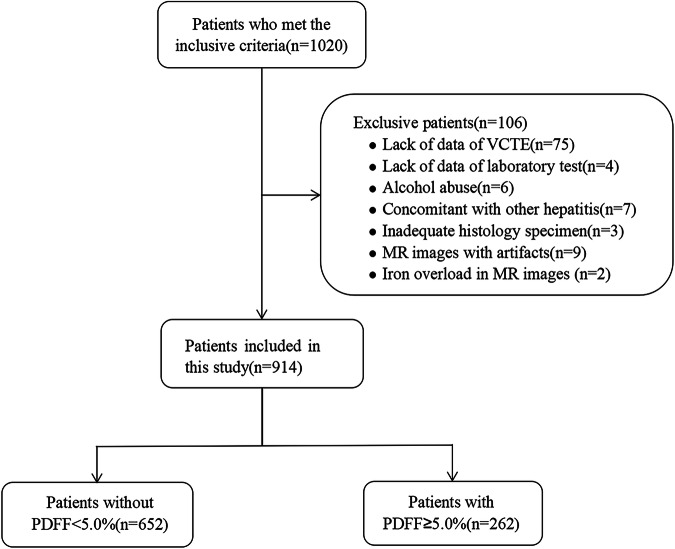
Flowchart of patient inclusion and exclusion

Of 1020 consecutive patients with CHB who underwent CSE-MRI and liver biopsies during the study period, 914 were enrolled in this study (Fig. 1). Their median age was 41.0 (IQR, 35.0–49.0) years, 579 (63.3%) were male, 165 (18.1%) had hypertension, and 202 (22.1%) had DM or IGT. The median time interval between CSE-MRI and liver biopsy was 0 days (range: 0–30 days), with 81.5% of MRIs being performed on the same day as liver biopsy. The median time interval between VCTE and liver biopsy was 2 days (range: 0–30 days).

According to the MRI-PDFF results, 262 (28.7%) patients had hepatic steatosis. The median MRI-PDFF values in patients with non-steatosis and steatosis were 3.34% and 7.10%, respectively. The clinicobiochemical characteristics of the two groups are shown in Table 1.

**Table 1:** Clinical characteristics of CHB patients with non-steatosis and steatosis based on MRI-PDFF

Characteristic	Non-steatosis, (*n* = 652)	Steatosis, (*n* = 262)	*p* value
Age, (year)	41.0 (35.0–49.0)	41.0 (35.0–50.0)	0.798
Gender			< 0.001
Female	285 (43.6%)	51 (19.5%)	
Male	369 (56.4%)	211 (80.5%)	
Hypertension	92 (14.1%)	53 (20.2%)	0.022
DM or IGT	32 (4.9%)	39 (14.9%)	< 0.001
BMI, (kg/m^2^)	22.3 (20.3–24.3)	25.0 (22.9–27.3)	< 0.001
PLT, (× 10^9^/L)	202 (173–244)	209 (180–244)	0.208
ALT, (U/L)	24.0 (17.0–41.0)	33.0 (24.0–51.0)	< 0.001
AST, (U/L)	23.0 (18.0–33.0)	27.0 (22.0–40.0)	< 0.001
ALB, (g/L)	45.6 (42.8–47.8)	46.3 (43.4–48.2)	0.063
ALP, (U/L)	67.0 (54.0–81.0)	71.0 (60.0–85.0)	0.002
GGT, (U/L)	22.0 (15.0–36.0)	33.0 (23.0–51.0)	< 0.001
TBIL, (μmol/L)	11.2 (8.7–14.9)	11.4 (9.3–14.9)	0.269
TC, (mmoL/L)	4.73, (4.11–5.30)	4.82 (4.18–5.51)	0.074
TG, (mmoL/L)	0.97 (0.73–1.27)	1.31 (0.98–1.82)	< 0.001
HDL-C, (mmoL/L)	1.31 (1.09–1.56)	1.07 (0.91–1.30)	< 0.001
LDL-C, (mmoL/L)	2.97 (2.48–3.53)	3.20 (2.60–3.71)	0.001
HBsAg, (COI)	2104 (1344–5776)	2144 (1363–6129)	0.634
HBeAg, (+)	285 (28.7%)	71 (27.1%)	0.631
HBV DNA, (IU/mL)	6210 (166–453,000)	5790 (119–572,500)	0.762
HBV DNA, (> 20,000 IU/mL)	270 (41.4%)	109 (41.6%)	0.958
Antivirus therapy	85 (13.0%)	35 (13.4%)	0.896
APRI	0.29 (0.21–0.47)	0.33 (0.24–0.49)	0.004
FIB-4	0.96 (0.71–1.40)	0.92 (0.66–1.33)	0.341
PDFF, (%)	3.34 (2.40–3.97)	7.10 (5.50–7.58)	< 0.001
Fibrosis staging			0.519
F0–1	342 (52.5%)	129 (49.2%)	
F2	196 (30.1%)	76 (29.0%)	
F3	78 (12.0%)	39 (14.9%)	
F4	36 (5.5%)	18 (6.9%)	
Necroinflammatory activity grading			0.408
G0–1	358 (54.9%)	137 (52.3%)	
G2	229 (35.1%)	98 (37.4%)	
G3	55 (8.4%)	26 (9.9%)	
G4	10 (1.5%)	1 (0.4%)	

The chi-square test or Fisher exact probability test, as appropriate, was used to compare the difference in categorical variables among different groups. Mann–Whitney *U*-test was used to compare the difference in continuous variables. Numbers in parentheses represent percentages or IQR

*DM* diabetes mellitus, *IGT* impaired glucose tolerance, *BMI* body mass index, *PLT* platelets, *ALT* alanine aminotransferase, *AST* aspartate aminotransaminase, *ALB* albumin, *ALP* alkaline phosphatase, *GGT* gamma-glutamyl transferase, *TBIL* total bilirubin, *TC* total cholesterol, *TG* total triglyceride, *HDL-C* high-density lipoprotein cholesterol, *LDL-C* low-density lipoprotein cholesterol, *HBsAg* hepatitis B surface antigen, *HBeAg* hepatitis B e antigen, *HBV* *DNA* hepatitis B viral DNA, *APRI* AST to platelet ratio index, *FIB-4* fibrosis-4 index, *PDFF* proton density fat fraction

### Impact of steatosis on LSM values

As shown in Table 2, liver MRI-PDFF was significantly associated with LSM in the univariate linear regression analysis of the entire study cohort, with this significant association remaining after adjusting for potential confounders (age, sex, BMI, ALT, TBIL, antiviral therapy, HBeAg status, liver fibrosis stage, and necroinflammatory activity grade).

**Table 2:** Univariable and multivariable linear regression analysis of the effect of different variables on the LSM value in the entire cohort

	Univariable analysis			Multivariable analysis		
	*B*-coefficient	95% CI	*p* value	*B*-coefficient	95% CI	*p* value
Gender	0.051	0.035–0.066	< 0.001	0.021	0.008–0.034	0.001
Age	0.000	− 0.001 to 0.001	0.960	0.017	–	0.542
Antivirus	0.011	0.000–0.023	0.054	0.048	–	0.064
HBeAg positive	0.035	0.019–0.052	< 0.001	0.016	0.002–0.030	0.021
Necroinflammatory activity	0.083	0.073–0.092	< 0.001	0.028	0.016–0.040	< 0.001
Fibrosis stage	0.067	0.061–0.073	< 0.001	0.050	0.042–0.058	< 0.001
ALT	0.000	0.000–0.000	< 0.001	0.000	0.000–0.000	0.019
TBIL	0.003	0.002–0.004	< 0.001	0.048	–	0.086
PDFF	0.005	0.003–0.007	< 0.001	0.003	0.002–0.005	< 0.001
BMI	0.006	0.004–0.008	< 0.001	0.003	0.001–0.005	0.004

*CI* confidential interval

In the F0, F1, and F2 cohorts, liver MRI-PDFF was significantly associated with LSM in the univariate linear regression analysis. After adjusting for potential confounders (age, sex, BMI, ALT, TBIL, antiviral therapy, HBeAg status, and necroinflammatory activity grade), liver MRI-PDFF remained significantly associated with LSM in the F1 and F2 cohorts, but not in the F0 cohort (Supplementary Table S1). Liver steatosis was not significantly associated with LSM in the F3 or F4 cohorts according to the univariate linear regression analyses.

### LSM in patients with different degrees of hepatic steatosis

**Fig. 2:**
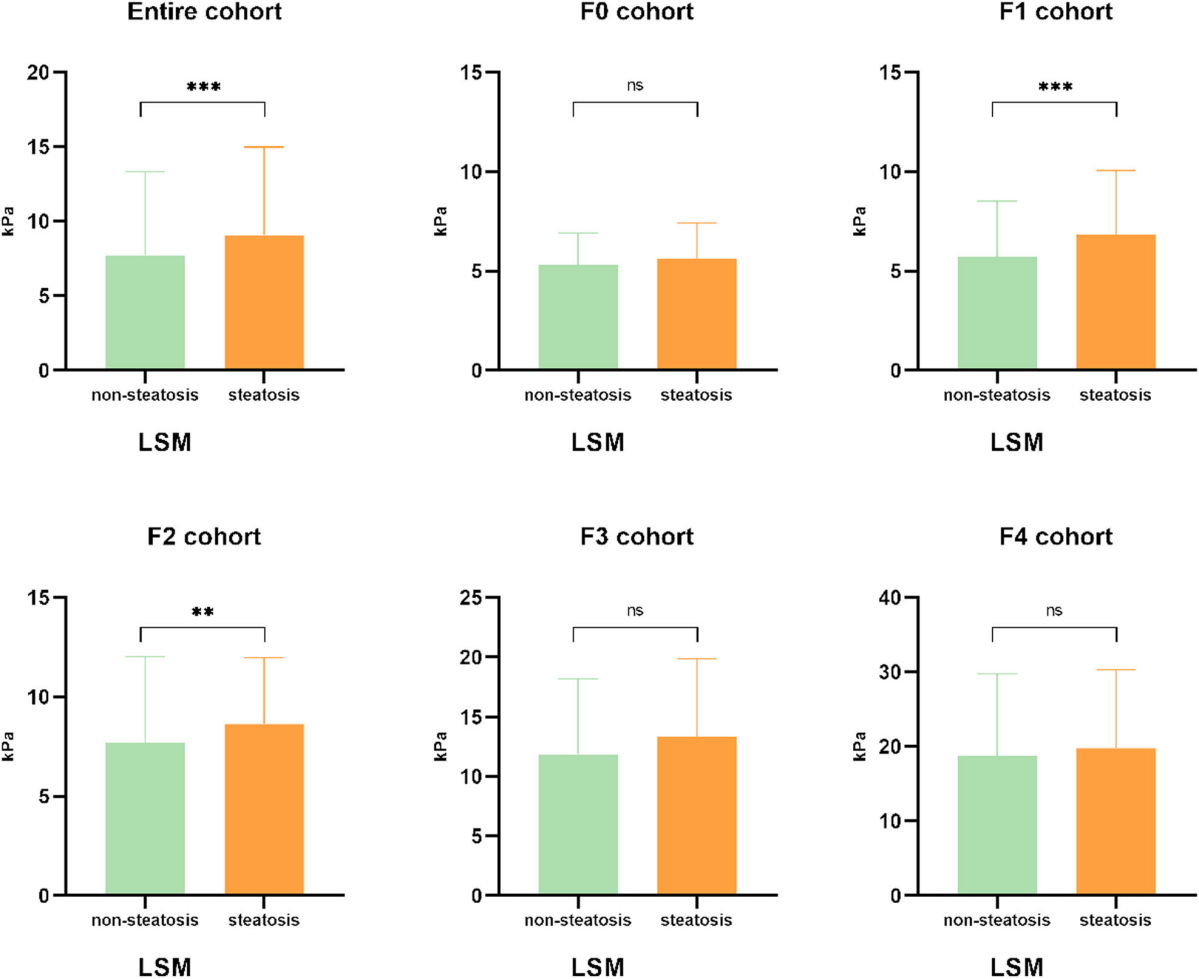
Distribution of LSM values between CHB patients with and without hepatic steatosis in the entire cohort and each fibrosis stage. For the entire CHB cohort, F1 cohort, and F2 cohort, the LSM values were significantly higher in CHB patients with hepatic steatosis than in those without hepatic steatosis. For F0, F3, and F4 cohorts, no significant difference was observed between patients with and without steatosis. **p < 0.01, ***p < 0.001

As shown in Fig. 2 and Table 3, LSM values differed significantly between CHB patients with and without steatosis in the entire, F1, and F2 cohorts but not in the F0, F3, and F4 cohorts. Similar findings were observed between patients with and without steatosis according to histology (Supplementary Tables S2 and 3 and Fig. S2).

**Table 3:** LSM values (kPa) of different liver fibrosis stages in CHB patients with non-steatosis and steatosis based on MRI-PDFF

	Non-steatosis	Steatosis	*p* value*
Entire cohort	6.00 (4.70–8.30)	7.30 (5.30–10.60)	0.001
F0 cohort	5.05 (4.10–6.15)	5.30 (4.60–6.20)	0.540
F1 cohort	5.00 (4.30–6.30)	5.95 (4.70–7.85)	< 0.001
F2 cohort	6.80 (5.40–8.40)	7.95 (6.05–10.90)	0.003
F3 cohort	10.45 (7.80–15.10)	11.10 (9.25–17.45)	0.177
F4 cohort	16.20 (10.55–24.60)	19.05 (11.30–26.60)	0.646
*p* value**	< 0.001	< 0.001	

Mann–Whitney *U*-test was used to compare the difference in continuous variables. The number in parentheses represents IQR

* Represented the *p* value for comparing the difference of LSM between non-steatosis cohort and steatosis cohort s in each liver fibrosis stages

** Represented the *p* value for comparing the difference of LSM between four cohorts with diverse liver fibrosis stages in a non-steatosis cohort or steatosis cohort

### Influence of hepatic steatosis on the diagnostic performance of LSM

**Fig. 3:**
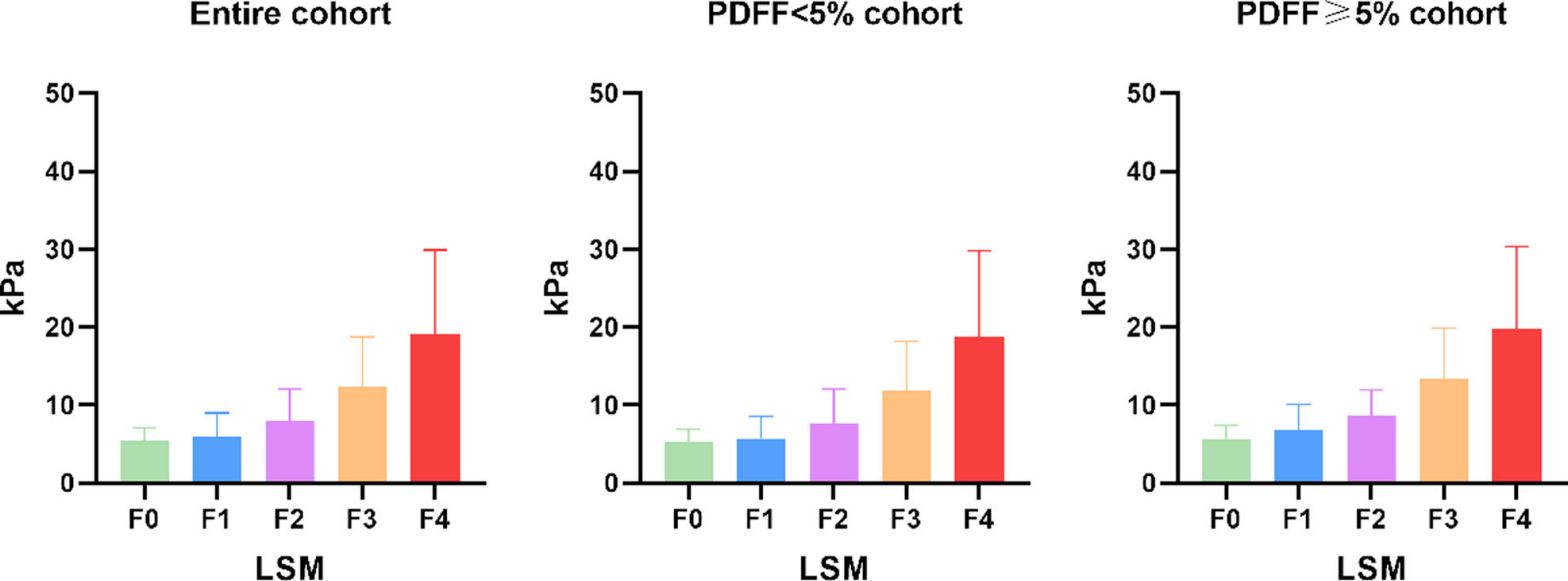
Distribution of LSM values between different liver fibrosis stages in the entire cohort, PDFF < 5% cohort, and MRI-PDFF ≥ 5% cohort. LSM values showed a significant increase as the liver fibrosis stage progressively increased (all *p* < 0.001)

**Fig. 4:**
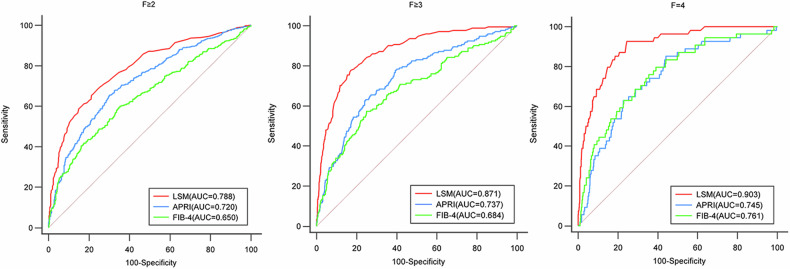
ROC curves of LSM, APRI, and FIB-4 for identifying *F* ≥ 2, *F* ≥ 3, and *F* = 4. LSM showed significantly better performance than APRI (all *p* < 0.001) and FIB-4 (all *p* < 0.001)

As shown in Fig. 3, the LSM values showed a significant increase as the liver fibrosis stage progressively increased in the entire cohort, non-steatosis cohort, and steatosis cohort. For all patients, the AUCs of LSM for identifying *F* ≥ 2, *F* ≥ 3, and *F* = 4 were 0.788, 0.871, and 0.903, respectively, showing significantly better performance than FIB-4 (all *p* < 0.001) and APRI (all *p* < 0.001) (Fig. 4).

**Fig. 5:**
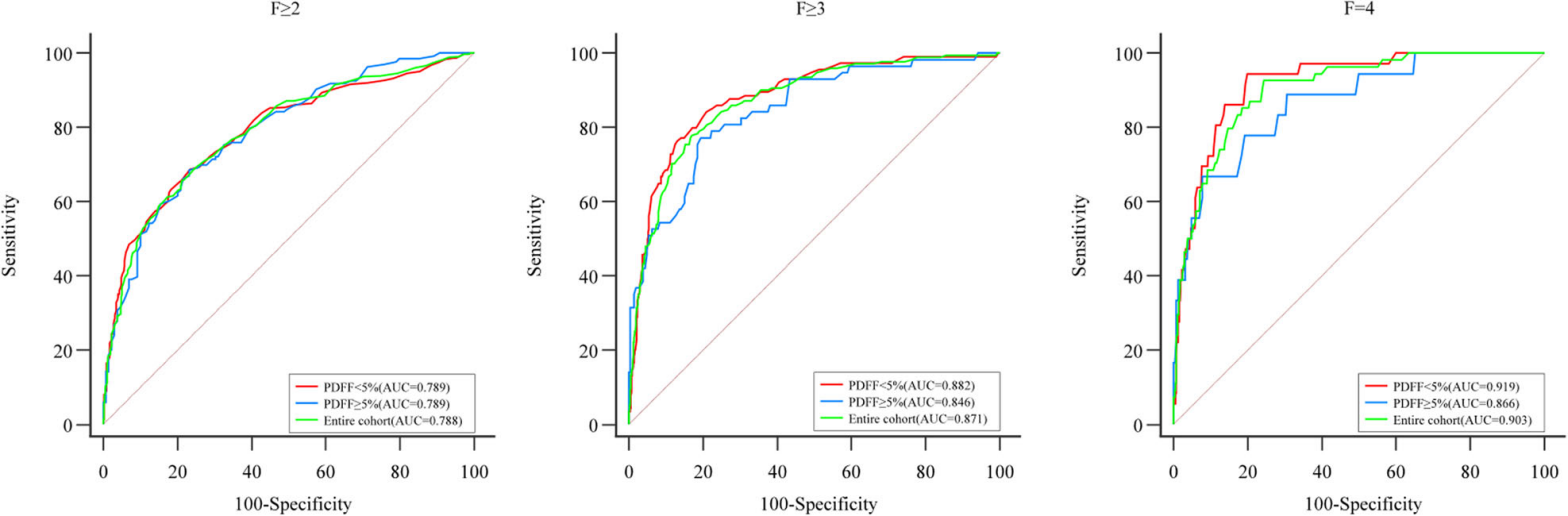
ROC curves of LSM for identifying *F* ≥ 2, *F* ≥ 3, and *F* = 4 in the entire cohort, MRI-PDFF < 5% cohort, and MRI-PDFF ≥ 5% cohort. No significant difference in the AUCs of LSM for identifying liver fibrosis stage was observed between those three cohorts

The influence of hepatic steatosis on the diagnostic performance of LSM was evaluated by stratifying patients according to their MRI-PDFF values (entire cohort, PDFF < 5% cohort, and PDFF ≥ 5% cohort). Hepatic steatosis showed no significant influence on the AUCs of LSM for identifying *F* ≥ 2, *F* ≥ 3, and *F* = 4. Details of AUCs comparisons are shown in Table 4 and Fig. 5. Similar findings were observed when patients were stratified according to histology results (entire cohort, S0 cohort, and S1–3 cohort) (Supplementary Table S4 and Figs. S3 and 4).

**Table 4:** Diagnostic performance of LSM for identifying *F* ≥ 2, *F* ≥ 3, and *F* = 4 by stratifying patients according to MRI-PDFF values

	*F* ≥ 2	*F* ≥ 3	*F* = 4
Stratum 1 AUROC (95% CI) for enter cohort	0.788 (0.760–0.814)	0.871 (0.847–0.892)	0.903 (0.882–0.921)
	Prevalence = 0.485 (443/914)	Prevalence = 0.187 (171/914)	Prevalence = 0.059 (54/914)
Stratum 2 AUROC (95% CI) for PDFF < 5.0	0.789 (0.755–0.819)	0.882 (0.855–0.906)	0.919 (0.895–0.939)
	Prevalence = 0.476 (310/652)	Prevalence = 0.175 (114/652)	Prevalence = 0.055 (36/652)
Stratum 3 AUROC (95% CI) for PDFF ≥ 5.0	0.789 (0.735–0.837)	0.846 (0.796–0.887)	0.866 (0.819–0.905)
	Prevalence = 0.508 (133/262)	Prevalence = 0.218 (57/262)	Prevalence = 0.069 (18/262)
AUROC comparison	Stratum 1/2: *p* = 0.990	Stratum 1/2: *p* = 0.630	Stratum 1/2: *p* = 0.561
	Stratum 1/3: *p* = 0.981	Stratum 1/3: *p* = 0.454	Stratum 1/3: *p* = 0.465
	Stratum 2/3: *p* = 0.990	Stratum 2/3: *p* = 0.296	Stratum 2/3: *p* = 0.294

The AUROC comparison was performed using the Delong test. The number in parentheses represents a 95% CI or positive cases divided by entire cases

### FPRs of different LSM cut-off values

As shown in Table 5, when using the LSM cut-off value of 7.0 kPa for identifying significant fibrosis (*F* ≥ 2), the FPR in the PDFF ≥ 5% cohort was higher than that in the PDFF < 5% cohort (*p* = 0.003); however, the sensitivity, PPV, NPV, and accuracy did not differ significantly between the two cohorts. Because the LSM cut-off value recommended by Duarte-Rojo et al for identifying *F* ≥ 2 in CHB patients is the same as that for hepatic steatosis patients, we attempted to assess the impact of an optimal cut-off value derived by Youden’s index on the FPR of LSM for identifying *F* ≥ 2 in CHB patients with concomitant steatosis (7.6 kPa). The results of this analysis showed that a relatively higher cut-off value could significantly lower the FPR of LSM in this cohort (*p* = 0.031), without significantly affecting its sensitivity, PPV, NPV, and accuracy (*p* > 0.05).

**Table 5:** The performance of LSM for identifying *F* ≥ 2, *F* ≥ 3, and *F* = 4 by stratifying patients according to MRI-PDFF values using different cut-off values

	PDFF < 5% cohort	PDFF ≥ 5% cohort	PDFF ≥ 5% cohort	*p* value*	*p* value**	*p* value***
*F* ≥ 2						
Cut-off, (kPa)	7.0	7.0	7.6			
Sensitivity, (%)	62.58	72.18	69.17	0.051	0.125	0.184
Specificity, (%)	82.16	69.77	74.42	**0.003**	**0.031**	0.061
FPR, (%)	17.84	30.23	25.58	**0.003**	**0.031**	0.061
PPV, (%)	76.08	71.11	73.60	0.285	0.654	0.599
NPV, (%)	70.78	70.87	70.07	0.985	0.395	0.294
Accuracy, (%)	72.85	70.99	71.76	0.570	0.754	0.737
*F* ≥ 3						
Cut-off, (kPa)	8.0	8.0	10.0			
Sensitivity, (%)	79.82	82.46	71.93	0.681	**0.031**	0.246
Specificity, (%)	82.90	68.29	81.46	**<** **0.001**	**<** **0.001**	0.645
FPR, (%)	17.10	31.71	18.54	**<** **0.001**	**<** **0.001**	0.645
PPV, (%)	49.73	41.96	51.90	0.195	0.175	0.747
NPV, (%)	95.10	93.33	91.26	0.403	0.482	0.063
Accuracy, (%)	82.36	71.37	79.39	**<** **0.001**	**<** **0.001**	0.295
*F* = 4						
Cut-off, (kPa)	11.0	11.0	13.0			
Sensitivity, (%)	72.22	77.78	66.7	0.751	0.500	0.673
Specificity, (%)	90.10	79.09	87.70	**<** **0.001**	**<** **0.001**	0.304
FPR, (100%)	9.90	20.90	12.30	**<** **0.001**	**<** **0.001**	0.304
PPV, (%)	29.89	21.54	28.57	0.248	0.408	0.878
NPV, (%)	98.23	97.97	97.27	0.764	0.755	0.405
Accuracy, (%)	89.11	78.6	86.26	**<** **0.001**	**<** **0.001**	0.225

McNemar’s test, chi-square test, or Fischer exact probability test, as appropriate, were used to compare the difference, data with statistically significant differences are indicated in bold

*FPR* false positive rate, *PPV* positive predictive value, *NPV* negative predictive value

* Represented the *p* value of PDFF < 5% cohort using low cut-offs vs PDFF ≥ 5% cohort using low cut-offs (7 kPa for *F* ≥ 2, 8 kPa for *F* ≥ 3, and 11 kPa for *F* = 4)

** Represented the *p* value of PDFF ≥ 5% cohort using high cut-offs (7.6 kPa for *F* ≥ 2, 10 kPa for *F* ≥ 3, and 13 kPa for *F* = 4) vs PDFF ≥ 5% cohort using low cut-offs (7 kPa for *F* ≥ 2, 8 kPa for *F* ≥ 3, and 11 kPa for *F* = 4)

*** Represented the *p* value of PDFF ≥ 5% cohort using high cut-offs (7.6 kPa for *F* ≥ 2, 10 kPa for *F* ≥ 3, and 13 kPa for *F* = 4) vs PDFF < 5% cohort using low cut-offs (7 kPa for *F* ≥ 2, 8 kPa for *F* ≥ 3, and 11 kPa for *F* = 4)

**Fig. 6:**
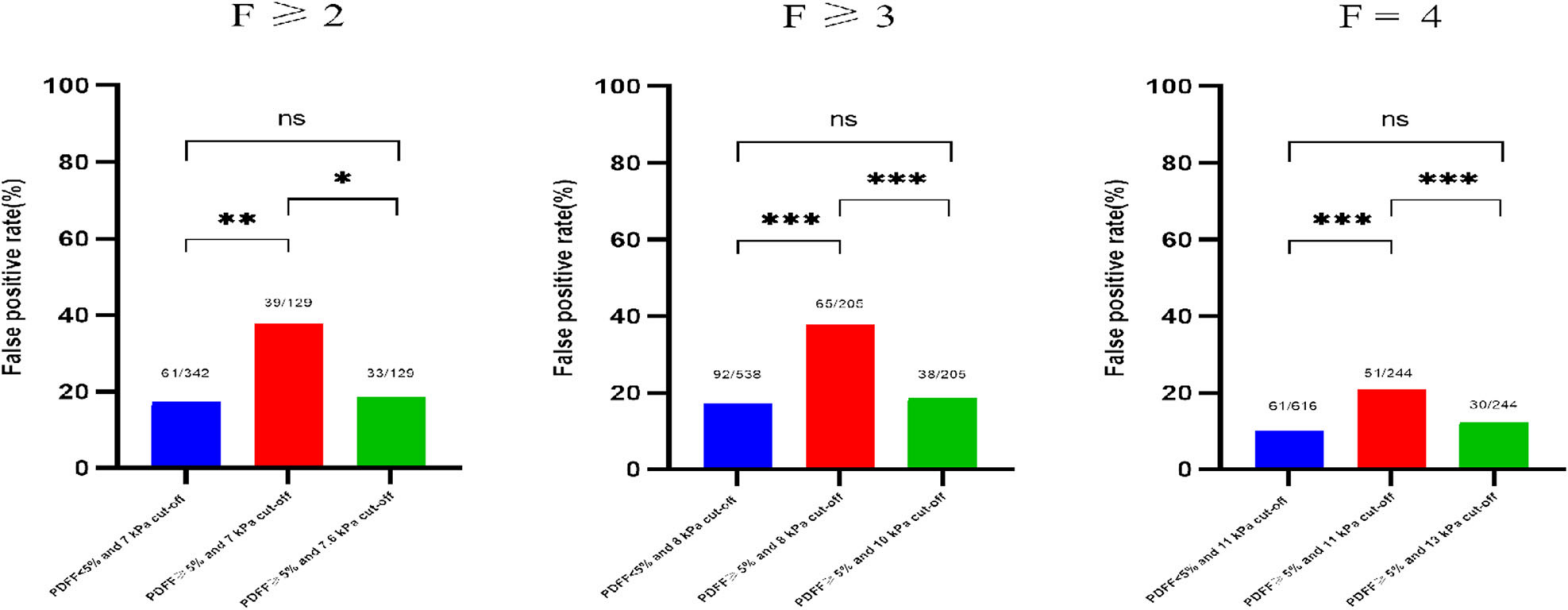
False-positive rates for the identification of significant fibrosis (*F* ≥ 2), advanced fibrosis (*F* ≥ 3) and cirrhosis (*F* = 4) were significantly higher in the steatosis cohort (red bar) than that in the non-steatosis cohort (blue bar) when using cut-off values for CHB patients (7 kPa for *F* ≥ 2, 8 kPa for *F* ≥ 3, and 11 kPa for *F* = 4). When higher cut-off values were used (7.6 kPa for *F* ≥ 2, 10 kPa for *F* ≥ 3, and 13 kPa for *F* = 4), false-positive rates in the steatosis cohort (green bar) were significantly decreased compared with those in the steatosis cohort using the cut-off values for CHB patients (red bar), and were comparable to those in patients without hepatic steatosis using the cut-off values for CHB patients (blue bar). *p < 0.05; **p < 0.01, and ***p < 0.001

When using the LSM cut-off values for the CHB cohort (8.0 kPa for *F* ≥ 3, and 11.0 kPa for *F* = 4) to identify advanced fibrosis and cirrhosis, the FPRs were significantly higher, and diagnostic accuracy was significantly lower in the PDFF ≥ 5% cohort than in the PDFF < 5% cohort (*p* < 0.001). Compared with using the cut-off values for the entire CHB cohort, the FPRs and accuracy of LSM for identifying advanced fibrosis and cirrhosis in the PDFF ≥ 5% cohort were significantly improved when the LSM cut-off values for the hepatic steatosis cohort were used (10.0 kPa for *F* ≥ 3, and 13.0 kPa for *F* = 4). Except for significantly decreased sensitivity for identifying *F* ≥ 3, the other LSM diagnostic performance metrics were not significantly different. Furthermore, the FPRs and accuracy of LSM in the PDFF ≥ 5% cohort when using cut-off values for the hepatic steatosis cohort were comparable to those in the PDFF < 5% cohort when using cut-off values for the CHB cohort (Fig. 6).

## Discussion

In our cohort of 914 consecutive patients with biopsy-proven CHB, we found that the VCTE-measured LSM value was significantly affected by steatosis in the early stage of liver fibrosis. However, hepatic steatosis was not observed to have any significant influence on the diagnostic performance of LSM. Moreover, compared with using the cut-off values for the CHB cohort, the FPR and specificity of LSM were significantly improved in the steatosis cohort when a relatively higher cut-off value was used for defining hepatic steatosis. These results should provide a basis for clinical decision-making.

With the increasing incidence of obesity and metabolic syndrome, concomitant MASLD is becoming common in patients with CHB, which increases the risk of fibrosis progression and a longer-term adverse prognosis [23–25]. Such patients require both antiviral therapy and lifestyle interventions, such as weight control and exercise. Thus, accurate assessment and dynamic monitoring of liver fat content are essential in this population. MRI-PDFF is currently the most accurate noninvasive method for measuring liver fat content, and is more sensitive than liver histology for assessing slight changes in liver fat. These advantages have ensured that MRI-PDFF has become one of the most preferred tools for fat quantification and longitudinal assessment in the clinical setting, especially for patients undergoing MRI for other reasons, such as ruling out cirrhosis, portal hypertension, or hepatic neoplasm.

Our study demonstrated that the LSM value increased as the liver fat content increased. However, this effect was slight, and was mainly manifested in the early stage of liver fibrosis. Indeed, no significant correlation was observed between steatosis and LSM in the high fibrosis cohort (*F* ≥ 3), which is in agreement with studies by Huang [26] and Liu [27]. Similar findings were also reported in a study conducted by Kumada et al [28], who found that LSM derived from two-dimensional shear wave elastography was significantly different across MRI-PDFF-based steatosis grades in patients with F0 and F1 fibrosis. In patients with steatosis, there is excess accumulation and enlargement of lipid droplets in the cytosol. These droplets are stiffer than the surrounding aqueous cytosol and may mechanically distort the hepatocytes and liver, potentially changing the propagation time of the vibratory wave through the liver (the key principle of VCTE) [25]. The correlation between liver elasticity and liver steatosis has been demonstrated in animal models subjected to elastography, and in studies on the biomechanical properties of both liver tissue and single hepatocytes [29]. However, as fibrosis progresses, compression of the excess extracellular matrix restricts the extension of cytosolic lipid droplets and alters important geometric parameters of cells, including cell height, surface extension, and cell contact area. This affects the biomechanical properties of lipid droplets and finally influences liver viscoelasticity. Furthermore, compared with the high elastic value in patients with advanced fibrosis or cirrhosis, which is increased by fibrosis collection, the effect of fatty changes on LSM was minimal in this study, and did not significantly affect the final measurement value.

Although significantly different LSM values were observed between patients with and without steatosis, the diagnostic efficiency for discriminating liver fibrosis showed no significant difference between patients with and without steatosis according to AUC values. This may be explained by the relatively uniform distribution of the degrees of steatosis across different liver fibrosis stages (Supplementary Tables S5–9 and Figs. S5–7). The effects of steatosis on LSM values were equal in each fibrosis group, and might not have significantly influenced diagnostic efficiency.

Although hepatic steatosis did not significantly affect the efficacy of VCTE for staging liver fibrosis, the LSM values were increased in low-grade fibrosis, resulting in an impact on the optimal cut-off values for staging liver fibrosis. A systematic review by Duarte-Rojo et al [22] showed that the cut-off values of VCTE for identifying advanced fibrosis and cirrhosis in MASLD patients were higher than those in CHB patients. Guidelines recommended different LSM cut-off values for individual diseases, but do not explicitly clarify which cut-off values should be used for patients with combined disease. At present, there are still no acknowledged LSM cut-off values for the staging of liver fibrosis in CHB patients with concomitant MASLD. Our study demonstrates that using cut-off values for staging of liver fibrosis that are intended for CHB patients would lead to higher FPRs in CHB patients with concomitant MASLD. Furthermore, the FPRs of LSM can be significantly improved by using the relatively higher cut-off values for hepatic steatosis patients, which suggests that the LSM cut-off values for the hepatic steatosis cohort were more suitable for the CHB patients with concomitant MASLD than the cut-off values for the CHB cohort.

This study has several limitations. First, despite the relatively large sample size, the number of cirrhosis patients with steatosis was relatively small. Thus, the results require confirmation in a larger multicenter prospective study. Second, PDFF was derived from either two- or six-point Dixon imaging, depending on availability (two-point Dixon, 60.2%). However, independent analyses of data for each technique showed similar results (Supplementary Tables 10–17 and Figs. S8–15), indicating that our conclusion was not affected by the use of different MRI vectors. Third, an XL probe was not used in our study because of equipment limitations, and obtaining data using an M probe was probably more difficult in some excessively obese patients, which could have led to bias in patient selection. Finally, we did not analyze the cost-efficiency of the examination because the availability and consequently the cost of VCTE and MRI-PDFF show worldwide geographical differences.

In conclusion, the results of our study indicated that steatosis diagnosed according to MRI-PDFF affected the VCTE-measured LSM, with a higher value being found in the early stage of liver fibrosis, but it did not affect the diagnostic efficiency of LSM for identifying liver fibrosis in patients with CHB. Moreover, in patients with CHB and an MRI-PDFF of ≥ 5%, the FPRs of LSM in the staging of liver fibrosis can be significantly improved by using cut-off values for hepatic steatosis patients rather than those for CHB patients.

## Supplementary information

ELECTRONIC SUPPLEMENTARY MATERIAL

## Data Availability

The data and material used and/or analyzed in this study are available from the corresponding authors upon reasonable request.

## Abbreviations

ALT: Alanine transaminase
APRI: AST to platelet ratio index
AST: Aspartate aminotransferase
BMI: Body mass index
CHB: Chronic hepatitis B
CI: Confidence interval
CSE: Chemical shift-encoded
DM: Diabetes mellitus
FIB-4: Fibrosis-4 index
FPR: False positive rate
HbeAg: Hepatitis B e antigen
HbsAg: Hepatitis B surface antigen
HBV DNA: Hepatitis B virus DNA
HBV: Hepatitis B virus
IGT: Impaired glucose tolerance
LSM: Liver stiffness measurement
MASLD: Metabolic dysfunction-associated steatotic liver disease
NPV: Negative predictive value
PDFF: Proton density fat fraction
PPV: positive predictive value
ROI: Region of interest
TBIL: Total bilirubin
VCTE: Vibration-controlled transient elastography
