# Supplementary material for: Impact of hepatic steatosis on liver stiffness measurement by vibration-controlled transient elastography and its diagnostic performance for identifying liver fibrosis in patients with chronic hepatitis B
Source: Insights Imaging. 2024 Nov 22;15:283. doi: 10.1186/s13244-024-01857-8 (PMC11584827; doi:10.1186/s13244-024-01857-8)
Supplement: Supplementary file 1 — ELECTRONIC SUPPLEMENTARY MATERIAL [file 13244_2024_1857_MOESM1_ESM.pdf]

Impact of hepatic steatosis on liver stiffness measurement by vibration-controlled transient elastography and its diagnostic performance for identifying liver fibrosis in patients with chronic hepatitis B

ELECTRONIC SUPPLEMENTARY MATERIAL

Supplementary Method S1. Acquisition protocol of CSE-MRI on two MR scans

MR parameter in Prisma 3.0T, Siemens Medical System: repetition time (TR)=9.0 ms; first echo time (TE)=1.10 ms with 6 echoes collected with ΔTE=1.23 ms; flip angle(FA)=4°; slice thickness=3.5 mm; field of view=38×31 cm; matrix=160×106 and acquisition time=15 seconds.

MR parameter in Titan 3.0T, Toshiba Medical System: TR=5.1 ms; TE=1.1 ms with 2 echoes collected with ΔTE=1.7 ms; flip angle(FA)=12°; slice thickness, 8 mm; field of view=38×40 cm; matrix=256×192 and acquisition time=16 seconds.

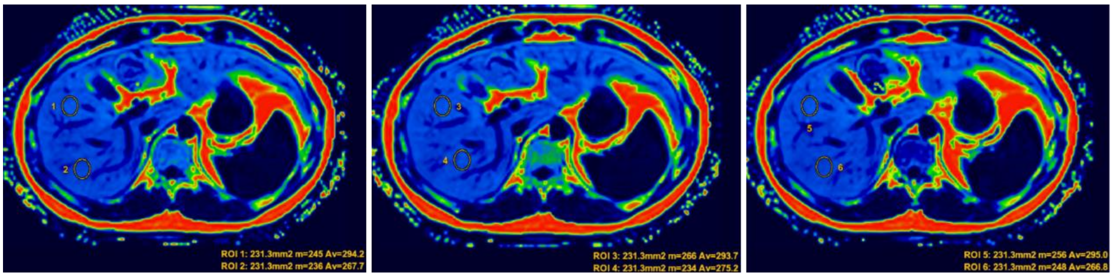

Figure S1. Diagram of ROIs on the fat fraction maps. Six 15-mm-diameter circular ROIs were placed in the right lobe on the central three continuous sections (two ROIs per section). the central layer was locate on where the right branch of the portal vein enters the liver. As showed in the bottom right corner, the ROIs area was approximately 231cm^2 and the average in each ROI value was the proportion of liver fat content multiplied by 10. The liver fat fraction of this patient was 28.21%.

Table S1. Univariable and multivariable linear regresion analysis of the effect of MRI-PDFF on the LSM value in entire cohort

	Univariable analysis			Multivariable analysis		
	B-coefficient	95% CI	*P* value	B-coefficient	95% CI	*P* value
Entire cohort	0.005	0.003-0.007	<0.001	0.003	0.002-0.005	<0.001
F0 cohort	0.004	0.001-0.009	0.039	0.118	-	0.335
F1 cohort	0.005	0.003-0.008	<0.001	0.004	0.002-0.007	<0.001
F2 cohort	0.004	0.001-0.007	0.002	0.003	0.001-0.006	0.002
F3 cohort	0.001	-0.004-0.007	0.593	-	-	-
F4 cohort	0.004	-0.002-0.010	0.206	-	-	-

Abbreviation: CI, confidential interval.

Table S2. LSM values(kPa) of different liver fibrosis stages in CHB patients with non-steatosis and steatosis by histology

	non-steatosis	steatosis	*P* value*
Entire cohort	6.10(4.75-8.50)	6.60(5.10-10.10)	0.013
F0 cohort	5.10(4.25-6.05)	5.40(4.30-6.20)	0.761
F1 cohort	5.00(4.30-6.60)	5.50(4.40-6.80)	0.029
F2 cohort	6.60(5.30-8.50)	7.80(6.20-10.40)	0.001
F3 cohort	10.45(7.75-15.15)	10.70(10.00-16.90)	0.306
F4 cohort	14.05(10.60-22.80)	21.55(12.65-27.00)	0.121
P value**	<0.001	<0.001	

Note: Mann-Whitney U test was used to compare the difference in continuous variables, Number in parentheses represent IQR.

* represented the *P* value for compared the difference of LSM between non-steatosis cohort and steatosis cohort s in each liver fibrosis stages.

** represented the *P* value for compared the difference of LSM between four cohorts with diverse liver fibrosis stages in non-steatosis cohort or steatosis cohort.

Table S3. LSM values(kPa) of different liver fibrosis stages in CHB patients with different grades of steatosis by histology

	S0	S1	S2	S3	*P* value*
Entire cohort	6.10 (4.75-8.50)	6.40 (4.90-10.00)	7.55 (6.10-10.80)	7.15 (5.70-11.15)	0.023
F0 cohort	5.10 (4.25-6.05)	4.75 (3.95-6.10)	6.55 (5.60-7.60)	-	0.057
F1 cohort	5.00 (4.30-6.60)	5.40 (4.40-6.65)	6.80 (5.20-7.90)	6.0	0.055
F2 cohort	6.60 (5.30-8.50)	7.70 (6.20-10.00)	8.30 (6.45-11.65)	6.85 (5.40-8.30)	0.010
F3 cohort	10.45 (7.75-15.15)	11.90 (10.10-16.90)	6.80 (6.25-8.65)	14.0	0.179
F4 cohort	14.05 (10.60-22.80)	21.30 (10.50-26.60)	34.80 (24.80-36.65)	-	0.144
P value**	<0.001	<0.001	0.030	0.407	

Note: Mann-Whitney U test was used to compare the difference in continuous variables, Number in parentheses represent IQR.

* represented the *P* value for compared the difference of LSM between non-steatosis cohort and steatosis cohort s in each liver fibrosis stages.

** represented the *P* value for compared the difference of LSM between four cohorts with diverse liver fibrosis stages in non-steatosis cohort or steatosis cohort.

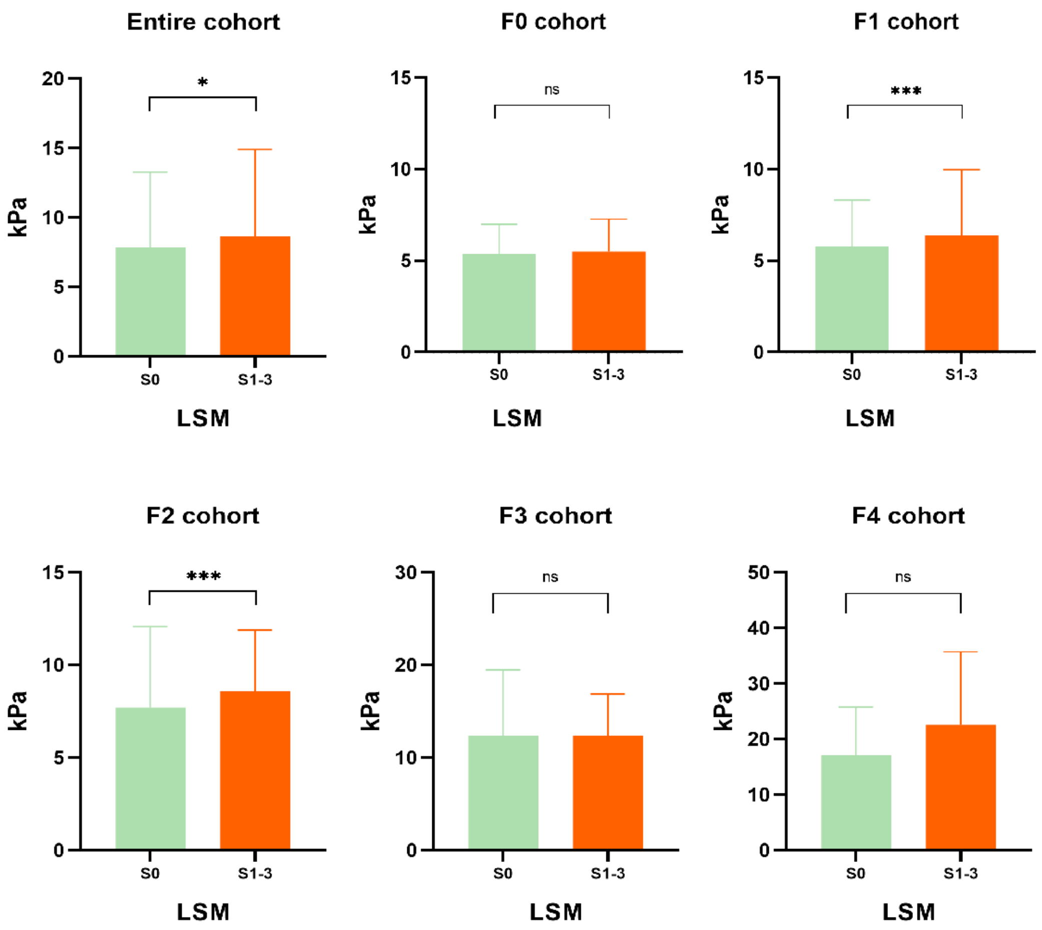

Figure S2. Distribution of LSM values between CHB patients with and without hepatic according to histological result (*represent P < 0.05, ***represent P < 0.001,^{ns}represent no significant).

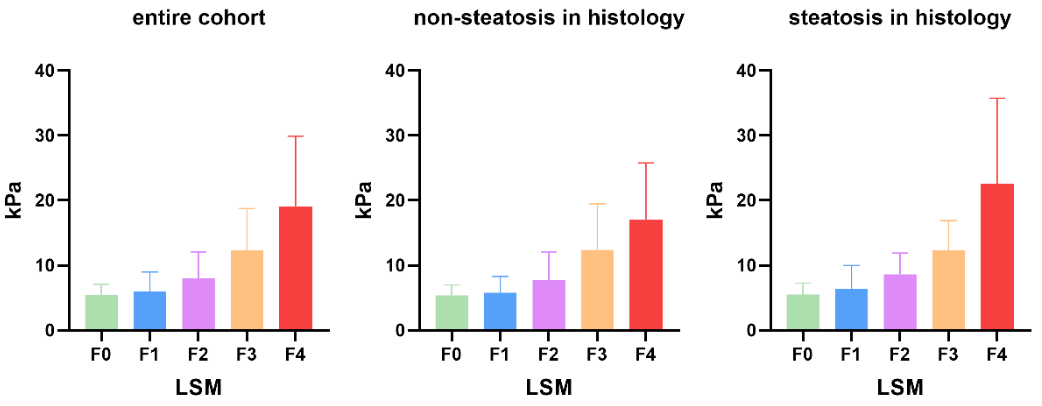

Figure S3. Distribution of LSM values between different liver fibrosis stages by stratifying patients according to histological results(all *P*<0.001).

Table S4. Diagnostic performance of LSM for identifying F ≥2, F≥3 and F=4 by stratifying patients according to histological results

	F≥2	F≥3	F=4
Stratum 1 AUROC (95%CI) for Enter cohort	0.788 (0.760-0.814)<br>Prevalence=0.485(443/914)	0.871 (0.847-0.892)<br>Prevalence=0.187(171/914)	0.903 (0.882-0.921)<br>Prevalence=0.059(54/914)
Stratum 2 AUROC (95%CI) for S0	0.775 (0.740-0.808)<br>Prevalence=0.507(308/607)	0.869 (0.839-0.895)<br>Prevalence=0.188(114/607)	0.897 (0.870-0.920)<br>Prevalence=0.056(34/607)
Stratum 3 AUROC (95%CI) for S1-3	0.831 (0.784-0.871)<br>Prevalence=0.440(135/307)	0.881 (0.840-0.915)<br>Prevalence=0.186(57/307)	0.914 (0.877-0.943)<br>Prevalence=0.065(20/307)
AUROC comparison	Stratum 1/2: P=0.584<br>Stratum 1/3: P=0.130<br>Stratum 2/3: P=0.066	Stratum 1/2: P=0.930<br>Stratum 1/3: P=0.733<br>Stratum 2/3: P=0.699	Stratum 1/2: P=0.858<br>Stratum 1/3: P=0.769<br>Stratum 2/3: P=0.684

Note: The AUROC comparison was performed using Delong test. Number in parentheses represent 95% confidential interval or positive cases divided by entire cases.

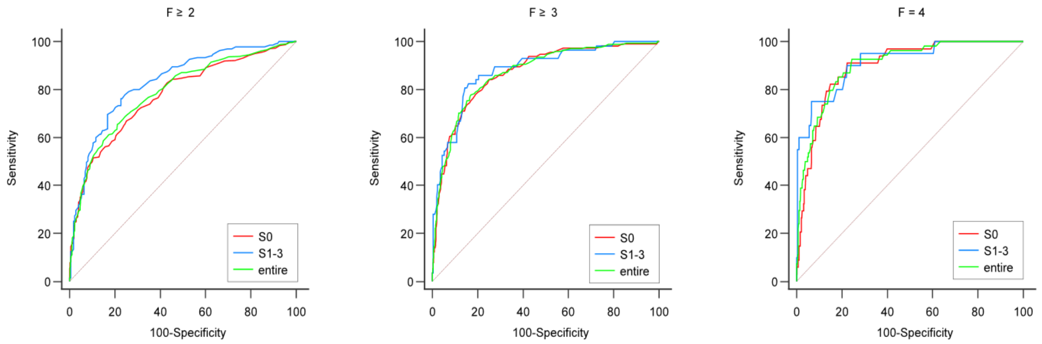

Figure S4. ROC curves of LSM, APRI, and FIB-4 for identifying F≥2, F≥3, and F=4 by stratifying patients according to histological results(all P>0.05).

Table S5. PDFF values(%) of different liver fibrosis stages in entire cohorts, 2-point Dixon cohort and 6-point Dixon cohort

	F0	F1	F2	F3	F4	*P* value
PDFF in entire cohort	3.56 (2.40-5.50)	3.56 (2.70-5.08)	3.80 (2.90-5.25)	4.20 (3.44-5.50)	4.28 (3.21-6.18)	0.010*
PDFF in 2-point Dixon cohort	4.28 (3.36-5.54)	4.04 (3.41-5.31)	4.32 (3.59-5.76)	4.41 (3.71-5.54)	4.70 (3.90-6.58)	0.141
PDFF in 6-point Dixon cohort	2.70 (1.40-4.90)	2.70 (1.70-4.55)	2.65 (1.50-4.00)	2.80 (1.90-4.75)	3.50 (2.10-5.20)	0.663

Note：*Kruskal-Wallis H test with Bonferroni post-test showed only a statistical difference between F1 and F3.

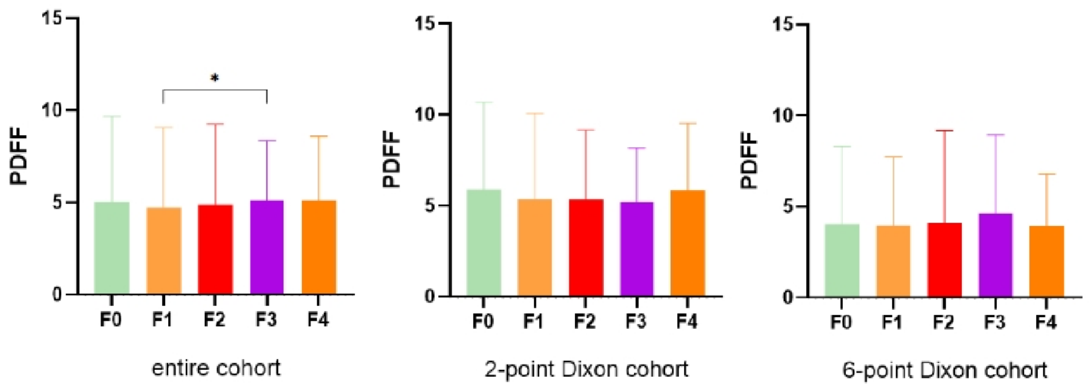

Figure S5. Distribution of PDFF values between different histological fibrosis stages in entire cohort, 2-point Dixon cohort and 6-point Dixon cohort (*represent $P < 0.05$).

Table S6. PDFF values of different liver fibrosis stages determined by VCTE-LSM in entire cohorts, 2-point Dixon cohort and 6-point Dixon cohort

	F0-1 (<7kPa)	F2 (7-8 kPa)	F3 (8-11 kPa)	F4 (≥11kPa)	P value
PDFF in entire cohort	3.57 (2.57-4.84)	3.70 (3.01-4.92)	4.30 (3.25-5.81)	4.49 (3.33-6.66)	<0.001*
PDFF in 2-point Dixon cohort	4.02 (3.42-5.29)	4.18 (3.47-4.92)	4.41 (3.62-5.80)	4.85 (3.70-6.82)	0.001**
PDFF in 6-point Dixon cohort	2.70 (1.60-4.00)	3.00 (1.70-4.70)	2.90 (2.00-5.90)	3.20 (1.80-6.30)	0.198

Note：*Kruskal-Wallis H test with Bonferroni post-test showed only a statistical difference for F0-1 vs F3 and F0-1 vs F4. **Kruskal-Wallis H test with Bonferroni post-test showed only a statistical difference between F0-1 and F4.

Table S7.PDFF values and LSM between different necroinflammatory activity grades in entire cohorts

	G0	G1	G2	G3	G4	P value
PDFF（%）	4.99 (4.42-5.56)	3.60 (2.70-5.26)	3.94 (3.10-5.26)	4.02 (3.30-5.36)	3.92 (1.95-4.41)	0.113
LSM（kPa）	4.50 (4.40-4.60)	5.40 (4.40-7.20)	7.30 (5.55-10.10)	13.90 (9.60-21.30)	17.60 (7.05-26.85)	<0.001

Table S8.PDFF values and LSM between different necroinflammatory activity grades in 6-point Dixon cohorts

	G1	G2	G3	G4	P value
PDFF（%）	2.20 (1.50-3.20)	2.25 (1.33-3.20)	2.90 (1.30-3.70)	1.95 (1.88- 2.10)	0.774
LSM（kPa）	6.65 (4.95-8.65)	6.65 (4.95-8.65)	15.20 (10.40-23.50)	20.00 (12.30-27.70)	<0.001

Table S9.PDFF values and LSM between different necroinflammatory activity grades in 2-point Dixon cohorts

	G0	G1	G2	G3	G4	P value
PDFF（%）	4.99 (4.42-5.56)	3.81 (3.33-4.66)	4.02 (3.48-4.93)	3.89 (3.35-4.74)	4.01 (3.92-4.11)	0.549
LSM（kPa）	4.50 (4.40-4.60)	5.45 (4.30-7.50)	7.55 (5.60-10.20)	11.80 (8.40-20.90)	6.85 (5.40-8.30)	<0.001

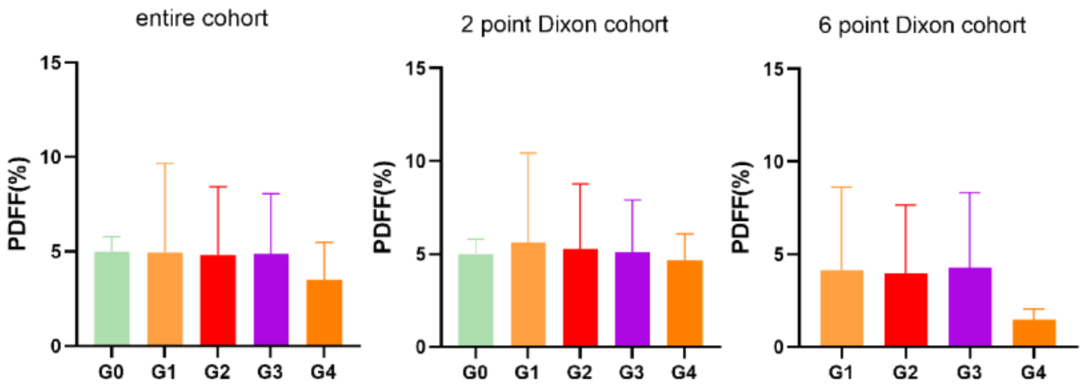

Figure S6. Distribution of PDFF values between different necroinflammatory activity grades in entire cohort, 2-point Dixon cohort and 6-point Dixon cohort (all P>0.05).

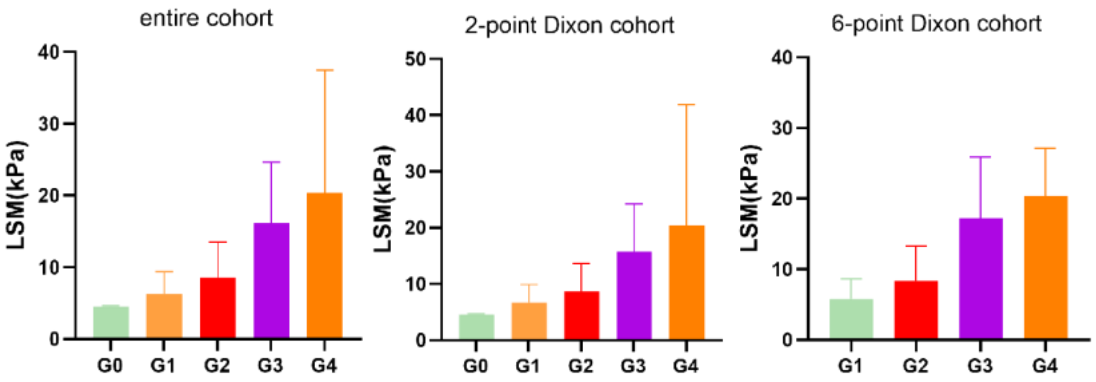

Figure S7. Distribution of LSM values between different necroinflammatory activity grades in entire cohort, 2-point Dixon cohort and 6-point Dixon cohort (all P<0.001).

Table S10. Univariable and multivariable linear regresion analysis of the effect of MRI-PDFF on the LSM value in patients using 6-point Dixon

	Univariable analysis			Multivariable analysis		
	B-coefficient	95% CI	*P* value	B-coefficient	95% CI	*P* value
Entire cohort	0.006	0.003-0.008	<0.001	0.005	0.003-0.008	<0.001
F0	0.006	-0.006-0.011	0.528	-	-	-
F1	0.007	0.003-0.010	<0.001	0.006	0.002-0.009	0.002
F2	0.004	0.001-0.008	0.015	0.004	0.001-0.008	0.024
F3	0.004	-0.010-0.017	0.592	-	-	-
F4	0.001	-0.009-0.011	0.206	-	-	-

Abbreviation: CI, confidential interval

Table S11. Univariable and multivariable linear regresion analysis of the effect of variables on the LSM value in 6-point Dixon cohort

	Univariable analysis			Multivariable analysis		
	B-coefficient	95% CI	P value	B-coefficient	95% CI	P value
Gender	0.044	0.019-0.068	0.001	0.083	-	0.051
Age	0.000	-0.001-0.002	0.587	-0.020	-	0.628
Antivirus	0.016	-0.002-0.035	0.085	0.015	0.000-0.030	0.043
HBeAg positive	0.018	-0.011-0.048	0.219	-0.003	-	0.948
Necroinflammatory activity	0.070	0.060-0.081	<0.001	0.045	0.026-0.045	<0.001
Fibrosis stage	0.067	0.061-0.073	<0.001	0.049	0.036-0.062	<0.001
ALT	0.001	0.000-0.001	<0.001	0.047	-	0.306
TBIL	0.003	0.002-0.005	<0.001	0.082	-	0.052
PDFF	0.004	0.002-0.007	0.003	0.005	0.002-0.007	<0.001
BMI	0.005	0.002-0.008	0.004	0.048	-	0.301

Abbreviation: CI, confidential interval.

Table S12. Univariable and multivariable linear regresion analysis of the effect of MRI-PDFF on the LSM value in patients using 2-point Dixon

	Univariable analysis			Multivariable analysis		
	B-coefficient	95% CI	*P* value	B-coefficient	95% CI	P value
Entire cohort	0.004	0.002-0.007	<0.001	0.004	0.002-0.006	0.001
F0	0.005	0.001-0.010	0.025	0.207	-	0.158
F1	0.005	0.002-0.008	0.002	0.005	0.002-0.008	0.003
F2	0.004	0.001-0.009	0.047	0.078	-	0.328
F3	0.001	-0.005-0.007	0.717	-	-	-
F4	0.008	0.000-0.017	0.061	-	-	-

Abbreviation: CI, confidential interval

Table S13. Univariable and multivariable linear regresion analysis of the effect of different variables on the LSM value in 2-point Dixon cohort

	Univariable analysis			Multivariable analysis		
	B-coefficient	95% CI	*P* value	B-coefficient	95% CI	*P* value
Gender	0.051	0.031-0.071	<0.001	0.023	0.006-0.040	0.007
Age	0.000	-0.001-0.001	0.845	0.032	-	0.383
Antivirus	0.009	-0.006-0.023	0.232	0.029	-	0.394
HBeAg positive	0.039	0.019-0.060	<0.001	0.024	0.007-0.041	0.005
Necroinflammatory activity	0.075	0.063-0.087	<0.001	0.022	0.008-0.037	0.002
Fibrosis stage	0.064	0.056-0.071	<0.001	0.049	0.039-0.058	<0.001
ALT	0.000	0.000-0.000	<0.001	0.000	0.000-0.000	0.025
TBIL	0.002	0.001-0.004	0.001	0.038	-	0.310
PDFF	0.004	0.002-0.007	<0.001	0.003	0.001-0.005	0.002
BMI	0.007	0.005-0.010	<0.001	0.004	0.002-0.006	0.001

Abbreviation: CI, confidential interval.

Table S14. LSM values(kPa) of different liver fibrosis stages in CHB patients with non-steatosis and steatosis based on MRI-PDFF in 6-point Dixon cohort

	non-steatosis	steatosis	*P* value*
Entire cohort	5.70(4.40-7.65)	6.65(5.15-9.85)	0.001
F0 cohort	5.20(3.80-6.10)	5.50(4.70-6.90)	0.540
F1 cohort	4.80(4.25-5.95)	5.80(5.00-7.80)	0.001
F2 cohort	6.20(5.10-8.10)	8.30(6.30-11.70)	0.017
F3 cohort	12.45(8.80-17.10)	11.85(6.70-18.00)	0.923
F4 cohort	17.60(13.75-24.60)	19.75(18.90-24.80)	0.733
P value**	<0.001	<0.001	

Note: Mann-Whitney U test was used to compare the difference in continuous variables, Number in parentheses represent IQR.

* represented the *P* value for compared the difference of LSM between non-steatosis cohort and steatosis cohort s in each liver fibrosis stages.

** represented the *P* value for compared the difference of LSM between four cohorts with diverse liver fibrosis stages in non-steatosis cohort or steatosis cohort.

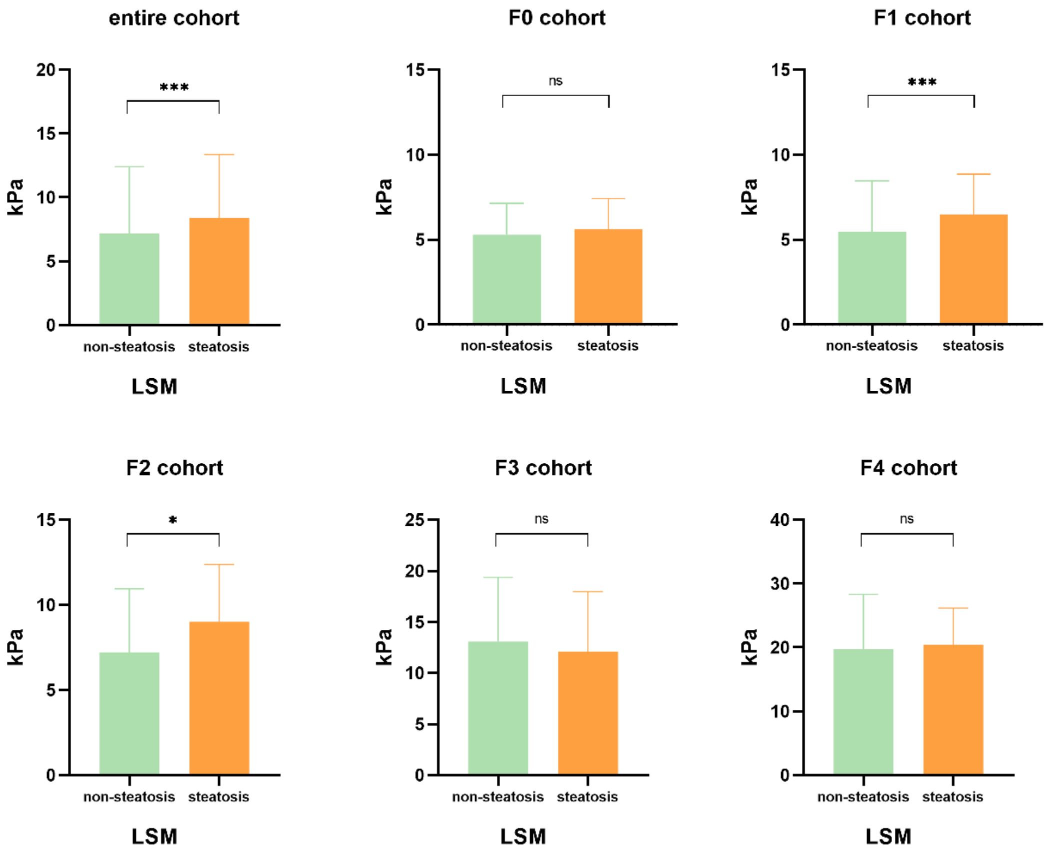

Figure S8. Distribution of LSM values in CHB patients with and without steatosis using 6-point Dixon(*represents $P < 0.05$, ***represents $P < 0.001$,^{ns}represents no significant).

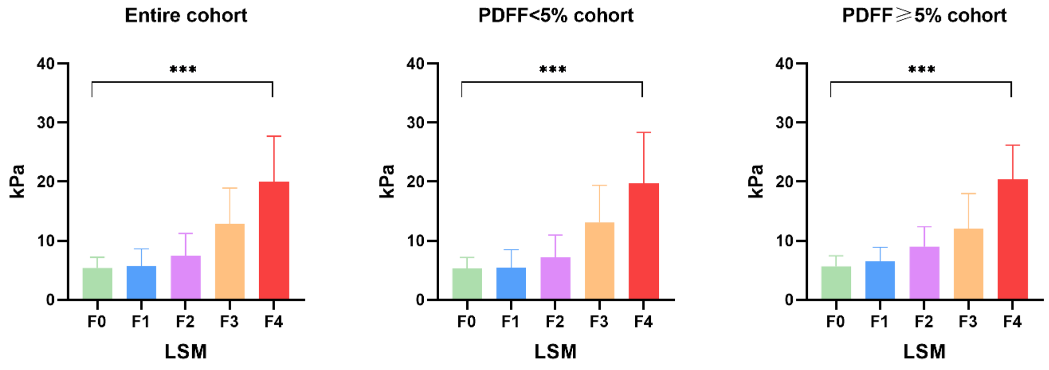

Figure S9. Distribution of LSM values between different liver fibrosis stages in CHB patients with and without steatosis using 6-point Dixon(***represents $P < 0.001$).

Table S15. LSM values(kPa) of different liver fibrosis stages in CHB patients with non-steatosis and steatosis based on MRI-PDFF in 2-point Dixon cohort

	**non-steatosis**	**steatosis**	***P* value***
Entire cohort	6.60(4.90-8.80)	7.50(5.50-11.20)	0.002
F0 cohort	5.0(4.40-6.20)	5.30(4.50-6.15)	0.891
F1 cohort	5.30(4.30-6.80)	6.00(4.65-7.95)	0.016
F2 cohort	7.00(5.50,8.80)	7.90(6.30-11.70)	0.094
F3 cohort	10.25(7.80,12.85)	11.10(9.80-16.90)	0.089
F4 cohort	14.20(10.50,22.80)	14.95(9.25-30.70)	0.897
P value**	<0.001	<0.001	

Note: Mann-Whitney U test was used to compare the difference in continuous variables, Number in parentheses represent IQR.

* represented the *P* value for compared the difference of LSM between non-steatosis cohort and steatosis cohort s in each liver fibrosis stages.

** represented the *P* value for compared the difference of LSM between four cohorts with diverse liver fibrosis stages in non-steatosis cohort or steatosis cohort.

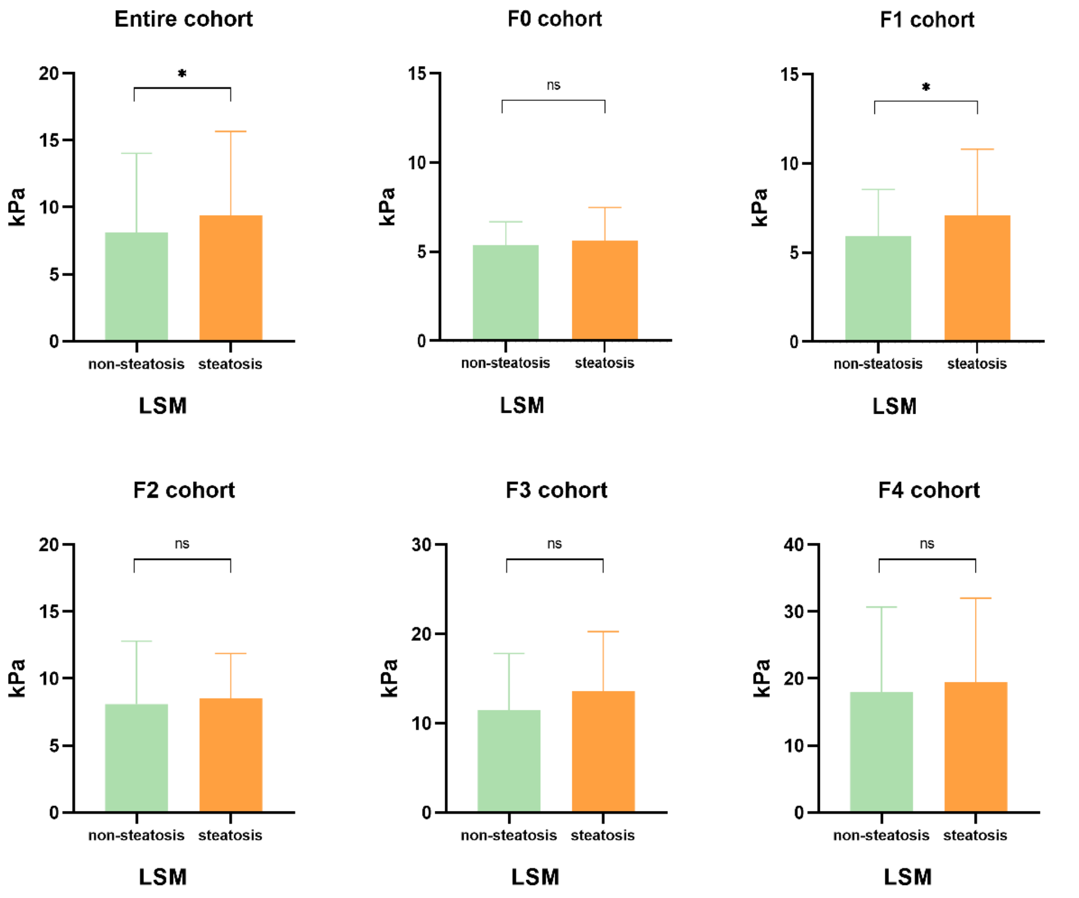

Figure S10. Distribution of LSM values in CHB patients with and without steatosis using 2-point Dixon(*represents $P < 0.05$, ^{ns}represents no significant).

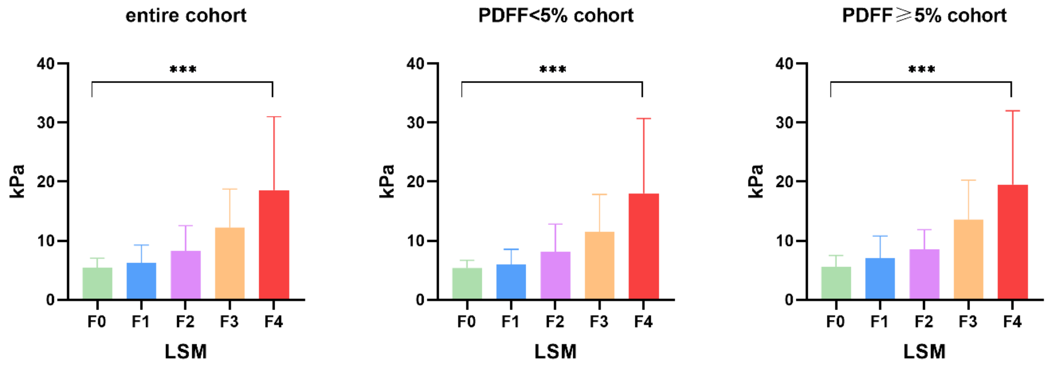

Figure S11. Distribution of LSM values between different liver fibrosis stages in CHB patients with and without steatosis using 2-point Dixon.

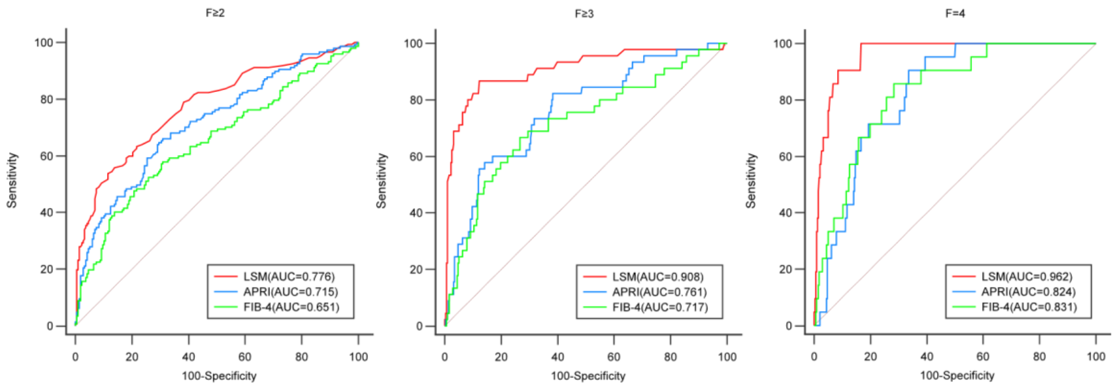

Figure S12. ROC curves of LSM, APRI, and FIB-4 for identifying F≥2, F≥3, and F=4 in CHB patients using 6-point Dixon.

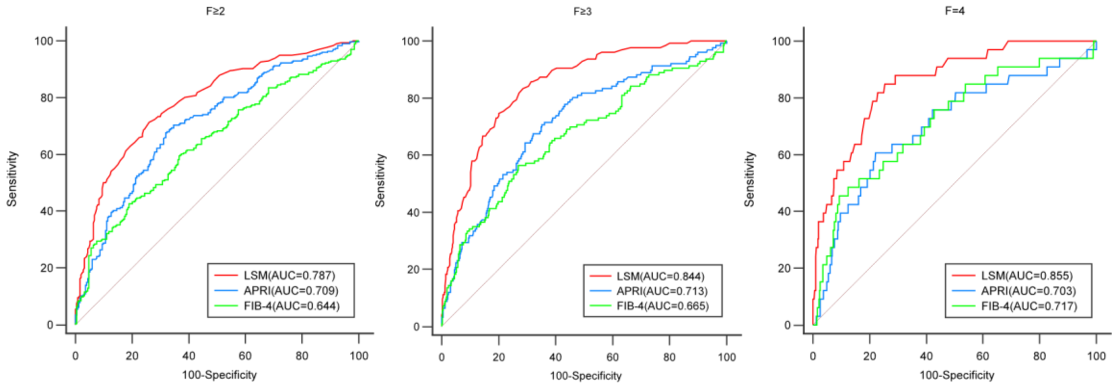

Figure S13. ROC curves of LSM, APRI, and FIB-4 for identifying F≥2, F≥3, and F=4 in CHB patients using 2-point Dixon(***represents $P < 0.001$).

Table S16. Diagnostic performance of LSM for identifying F ≥2, F≥3 and F=4 by stratifying patients according to MRI-PDFF values in patients using 6-point Dixon

	F≥2	F≥3	F=4
Stratum 1 AUROC (95%CI) for Enter cohort	0.776 (0.730-0.818)<br>Prevalence=0.404(147/364)	0.908 (0.873-0.936)<br>Prevalence=0.124(45/364)	0.962 (0.936-0.979)<br>Prevalence=0.058(21/364)
Stratum 2 AUROC (95%CI) for PDFF＜5.0	0.778(0.726-0.825)<br>Prevalence=0.416(118/284)	0.916 (0.878-0.946)<br>Prevalence=0.116(33/284)	0.961 (0.931-0.980)<br>Prevalence=0.053(15/284)
Stratum 3 AUROC (95%CI) for PDFF≥5.0	0.811 (0.708-0.890)<br>Prevalence=0.363(29/80)	0.876 (0.783-0.939)<br>Prevalence=0.150(12/80)	0.974 (0.911-0.997)<br>Prevalence=0.075(6/80)
AUROC comparison	Stratum 1/2: P=0.951<br>Stratum 1/3: P=0.553<br>Stratum 2/3: P=0.590	Stratum 1/2: P=0.854<br>Stratum 1/3: P=0.672<br>Stratum 2/3: P=0.604	Stratum 1/2: P=0.959<br>Stratum 1/3: P=0.636<br>Stratum 2/3: P=0.618

Note: The AUROC comparison was performed using Delong test.

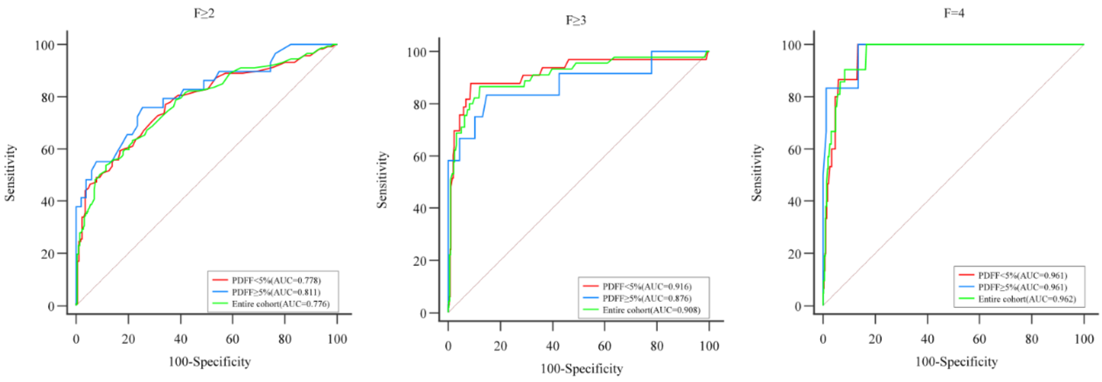

Figure S14. ROC curves of LSM for identifying F≥2, F≥3, and F=4 in PDFF<5% cohort, PDFF≥5% cohort and entire cohort by using 6-point Dixon.

Table S17. Diagnostic performance of LSM for identifying F ≥2, F≥3 and F=4 by stratifying patients according to MRI-PDFF values in patients using 2-point Dixon

	F≥2	F≥3	F=4
Stratum 1 AUROC (95%CI) for Enter cohort	0.787 (0.750-0.820)<br>Prevalence=0.538(296/550)	0.844 (0.811-0.873)<br>Prevalence=0.229(126/550)	0.855 (0.823-0.884)<br>Prevalence=0.060(33/550)
Stratum 2 AUROC (95%CI) for PDFF＜5.0	0.788 (0.743-0.829)<br>Prevalence=0.522(192/368)	0.854(0.813-0.888)<br>Prevalence=0.220(81/368)	0.881 (0.843-0.912)<br>Prevalence=0.057(21/368)
Stratum 3 AUROC (95%CI) for PDFF≥5.0	0.778 (0.711-0.837)<br>Prevalence=0.570(104/182)	0.831(0.769-0.883)<br>Prevalence=0.247(45/182)	0.811(0.746-0.865)<br>Prevalence=0.066(12/182)
AUROC comparison	Stratum 1/2: P=0.959<br>Stratum 1/3: P=0.837<br>Stratum 2/3: P=0.816	Stratum 1/2: P=0.753<br>Stratum 1/3: P=0.741<br>Stratum 2/3: P=0.590	Stratum 1/2: P=0.598<br>Stratum 1/3: P=0.553<br>Stratum 2/3: P=0.363

Note: The AUROC comparison was performed using Delong test.

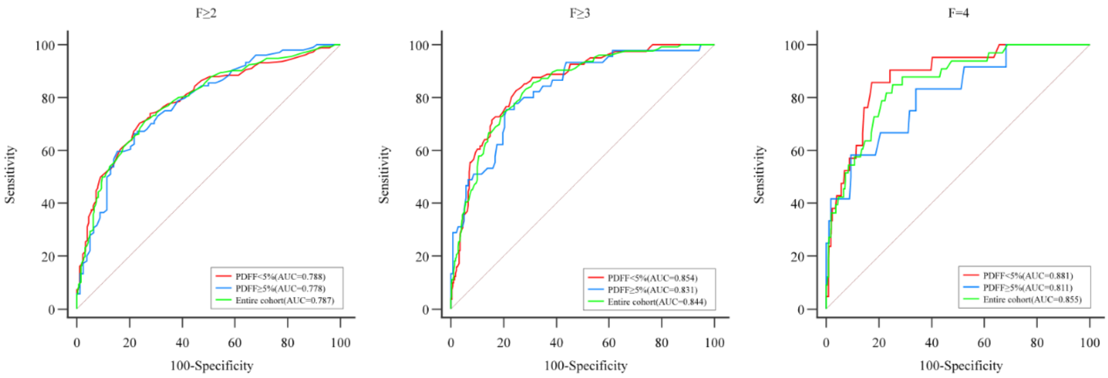

Figure S15. ROC curves of LSM for identifying F≥2, F≥3, and F=4 in PDFF<5% cohort, PDFF≥5% cohort and entire cohort by using 2-point Dixon.
